# Comparison of the efficacy and safety of holmium laser with the Moses technology and regular mode for stone treatment: a systematic review and meta-analysis

**DOI:** 10.1186/s12894-023-01264-z

**Published:** 2023-05-30

**Authors:** Puhan Li, Yucheng Ma, Chi Yuan, Zhongyu Jian, Xin Wei

**Affiliations:** Department of Urology, Institute of Urology (Laboratory of Reconstructive Urology), West China Hospital, Sichuan University, Chengdu, 610041 Sichuan China

**Keywords:** Moses technology, Holmium: YAG, Lasers, Lithotripsy, Urolithiasis, Meta-analysis

## Abstract

**Background:**

As a new pulse modality of holmium laser in retrograde intrarenal stone surgery, the MOSES technique can reduce the possibility of stone drifting and help to powder kidney stones in vitro and in animal experiments. However, there remains controversy about whether the MOSES mode needs to be used instead of the regular mode in clinical practice. This meta-review was conducted to evaluate the clinical efficacy and safety of MOSES technology for stone disease.

**Methods:**

PubMed, Embase, Web of Science, Cochrane Library, and CNKI were searched for relevant studies until September 2022, with 1 RCT and 6 nonrandomized studies included. We pulled data on adverse events, success rates and operative time to analyze based on the random effect model.

**Results:**

We found that using MOSES mode could shorten the operative time (standard mean difference [SMD] − 0.43; 95% confidence interval [CI] − 0.79 to  − 0.08; *P* = 0.016) than regular mode especially in a small sample study or in the Asian area. When the number of women is smaller than the number of men, the reduction of the duration was also significant. Stone-free rates of the two modes had no difference (relative risk [RR] 1.06; 95% CI 0.99–1.12; *P* = 0.30), and there was no publication bias. In terms of safety, no significant difference in complications was detected between the two approaches (RR 0.85; 95% CI 0.48–1.53; *P* = 0.81) without significant heterogeneity.

**Conclusion:**

MOSES mode holmium laser was superior to the regular mode laser in terms of procedure time. There was no large disparity in stone-free rates or complications between the two modes. However, our conclusions should be confirmed in prospective studies with high evidence.

**Supplementary Information:**

The online version contains supplementary material available at 10.1186/s12894-023-01264-z.

## Introduction

Nephrolithiasis is growing increasingly frequent around the world with an estimated prevalence in the U.S. of about 10.9% in men and 9.5% in women [1, 2]. As technology progresses, the use of the RIRS for the treatment of kidney calculi is increasingly accepted among urologists [3, 4]. Currently, the Holmium: YAG (Ho: TAG) laser lithotripsy has become the gold stand technique [5, 6], which can fragment or powder kidney stones [7]. Better surgical results can be achieved in less time with higher laser energy. However, high output and low-frequency holmium lasers can lead to low powdering efficiency, easily damaging the renal mucosa and causing stones to shift, which necessitated the use of pricey, delicate, flexible ureteroscopes to collect migrated stones [8].

To optimize the laser system in RIRS, a newly developed technique named MOSES mode has been introduced in kidney stone treatment. This method uses laser pulses first to separate the water, resulting in a short vapor bubble, and then transmit the remaining energy to the target stone with minimal energy loss. Many in vitro studies have shown that the MOSES mode can produce a greater ablation volume [9]. Fragmentation of more giant diameter stones results in a reduced rebound, reducing the chances of a stone moving to the kidneys, and increasing efficiency while minimizing ureteral tissue damage [10, 11]. Some clinical studies have been conducted recently and reported that MOSES mode could reduce operative time either [12–14]. However, there are little high-level researches comparing the duration, security, and effectiveness of two categories of holmium laser mode. To compare the MOSES and normal modalities of Holmium laser lithotripsy, this meta-analysis will pool published data to have a much more clear insight.

## Methods

### Search strategy and study selection

PubMed, Embase, Web of Science, Cochrane Library, and CNKI databases were searched to retrieve relevant literature with no linguistic constraints in the database until September 29, 2022. The search strategy was (((lithotripsy[Title/Abstract]) AND ((((laser[Title/Abstract]) OR (holmium laser[Title/Abstract])) OR (Holmium YAG Lasers[Title/Abstract])) OR (Lasers, Ho YAG[Title/Abstract]))) AND (Moses[Title/Abstract])) AND ((randomized controlled trial[Publication Type]) OR (prospective[Title/Abstract]) OR (retrospective[Title/Abstract]) OR (cohort study[Title/Abstract])). The other specific search strategies for the databases could be found in Additional file 1: Table S1.

The reference lists of the related articles were manually screened to ensure no missing data sources. The Preferred Reporting Items for Systematic Reviews and Meta-Analyses 2020 checklist was followed when conducting our systematic review [15]. This work was a review based on published data so that informed consent was not applicable.

Two authors independently compiled this work. The first phase was to screen the title and summary, while the second phase required evaluation of the full text. If there was disagreement between the authors, the corresponding author would make the decision.

Included studies must meet the following PICO inclusion criteria:

1. The patient had urinary stones that were treated with retrograde intrarenal stone surgery (RISR);
2. MOSES mode of holmium laser was the only intervention in treatment;
3. The comparison was between the MOSES mode and the regular mode;
4. Fundamental outcome information (e.g., stone-free rates, complications, and operation time) was included in the results;
5. RCTs, and prospective and retrospective studies were all pooled. If the studies had used previously published reviews and/or meta-analyses, they would be excluded from our meta-analysis. Moreover, all currently included articles were available in full text with sufficient data.

### Quality assessment

The Jadad scores were used to measure the performance of the RCTs included in the analysis. Two authors worked individually on the assessment method while the third author would resolve any disagreement. The Newcastle–Ottawa Scale (NOS) was used to estimate the probability of biases in non—randomized research.

### Data extraction and analysis

The goal of this meta-analysis was to investigate the efficacy and benefits of two holmium laser lithotripsy modalities in clinical use during ureteroscopy. The time of surgery and stone-removal performance were the leading indicators to evaluate the effectiveness. Since some studies did not mention the laser time, we chose procedure/operative time as the outcome. The primary comparison measure was the overall complication rate in both modes in terms of safety. Included papers provided information on the study strategy, publication year, an overall number of participants in each group (intervention and controls, stone-free or not, and overall cohort size), country of implementation, laser instrument types, and parameters. Two authors carried out and double-checked the data collection procedures independently.

Stata 14.0 (Stata Corp., College Station, TX, USA) was used to conduct the statistical analysis. Without any specific instructions, the results were statistically significant if two-tailed *P* < 0.05. The primary evaluation for continuous data was standard means difference (SMD) with a 95% confidence interval (95% CI), while the main estimate for discontinuous data was relative risk (RR) with a 95% CI. To quantify variability, the Q test and the I2 were utilized. When *P* < 0.05 in the Q test and I2 were larger than 50%, as well as a random effect model for pooling was applied, significant heterogeneity was identified. To determine the reason for heterogeneity and provide more information, sensitivity and subgroup analyses were utilized. Forest plots were built to emphasize the interesting conclusions. When Egger's tests were employed to examine publication bias, the funnel plot was removed as there are only seven papers included in the quantitative analysis. Any observed publication bias was recorded and analyzed using the trim-and-fill strategy to determine the impact of publication bias on the meta-analysis results.

## Results

### Search results

**Fig. 1:**
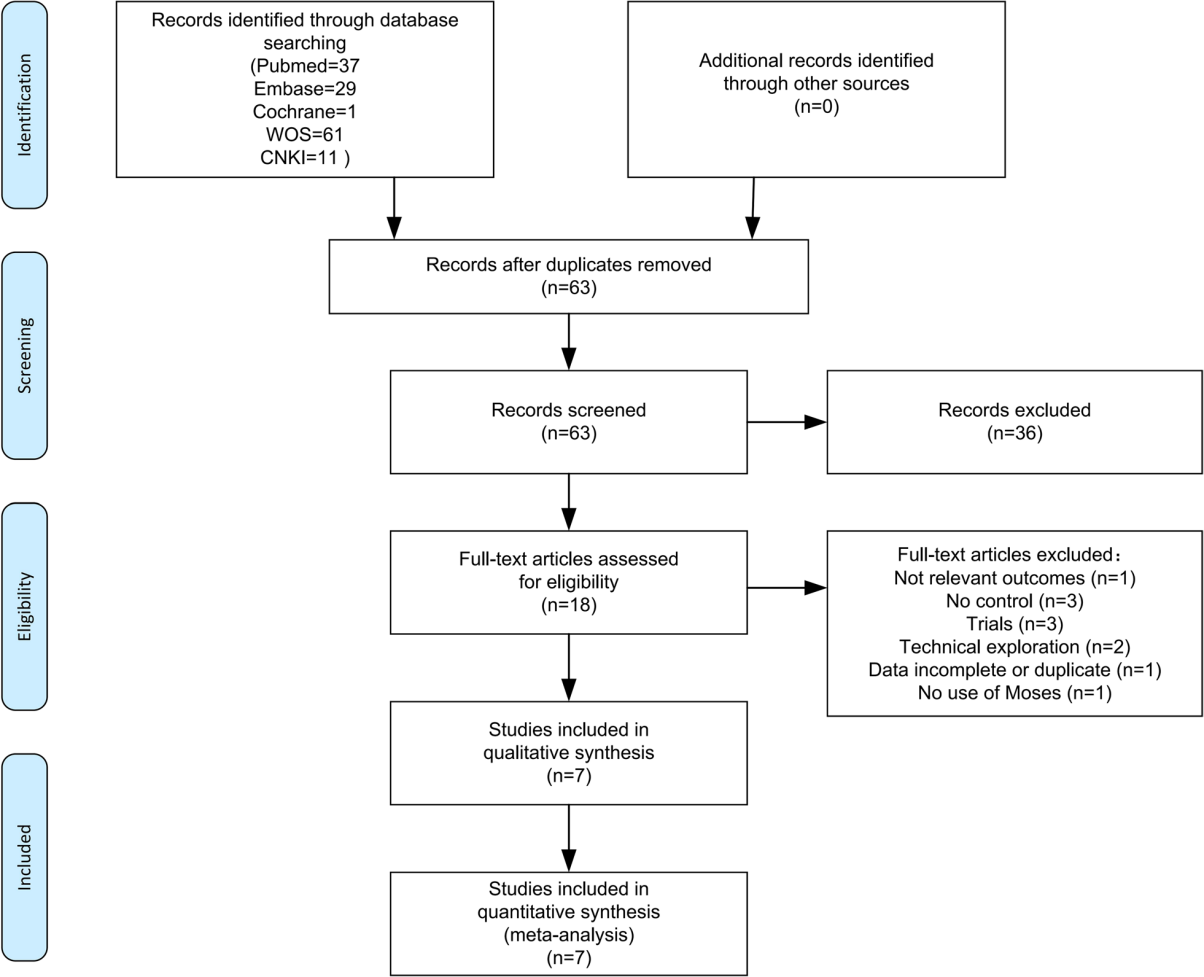
Selection flowchart

The original data retrieved from 1 RCT, 1 prospective cohort study (PCS), and 6 retrospective cohort studies (RCS) were included in this systematic review and meta-analysis after the literature screening and quality control [12–14, 16–19]. All of the studies contained efficacy and safety outcomes except the study from Harris W. N. etc., which didn’t have a detailed description of complications. There were 910 patients (398 for MOSES mode and 512 for Regular holmium laser) included in the quantitative analysis. Figure 1 illustrates the detailed flow chart. The studies selected in the analysis are listed in Additional file 2: Table S2. Furthermore, the results of the Jadad and NOS scales are shown in Additional files 3: Table S3 and 4: Table S4.

### Operative time comparison

**Fig. 2:**
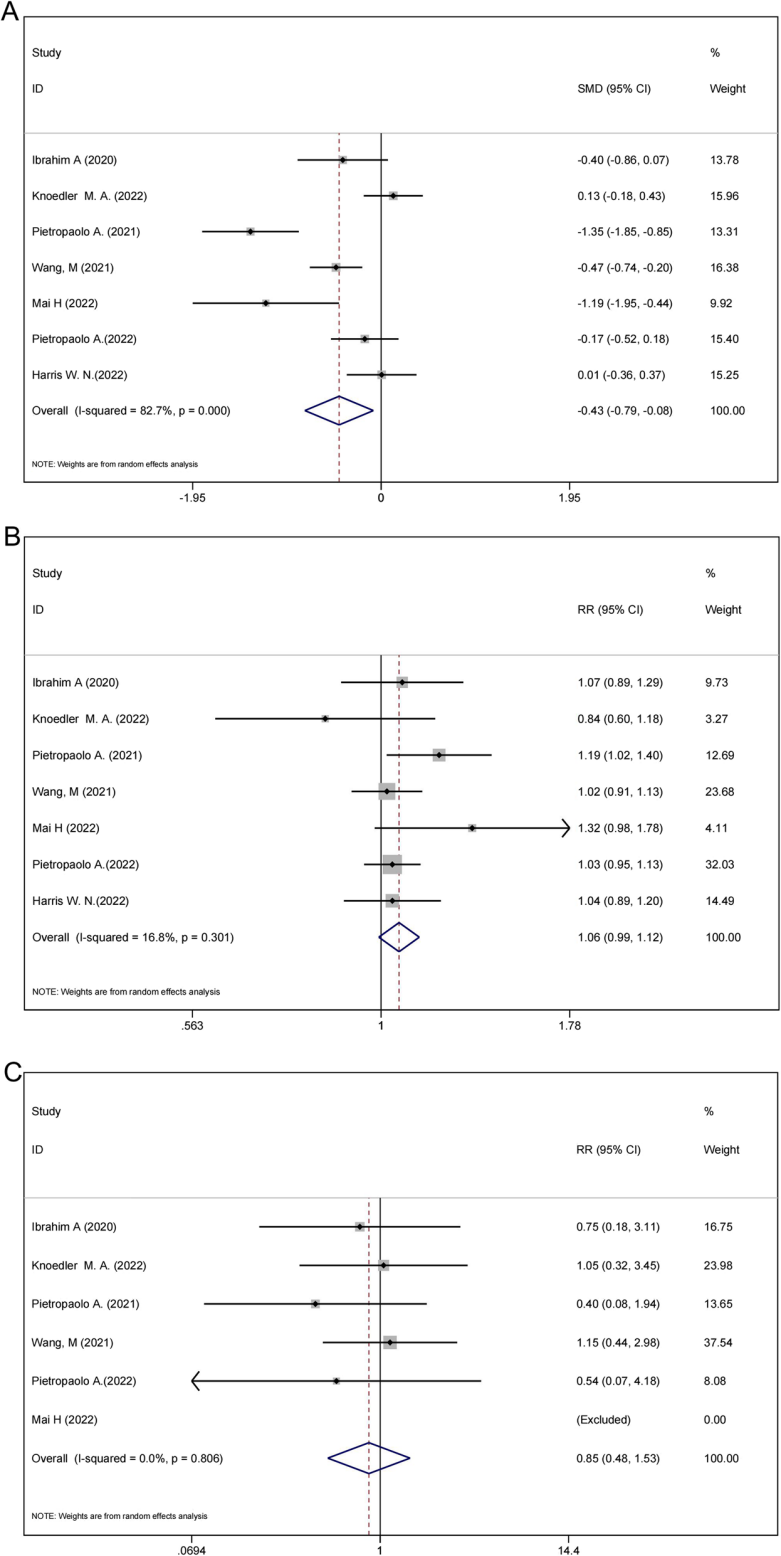
Forest plots of safety and efficacy comparisons between Moses mode and regular mode. **A** Operation time. **B** Stone-free rate. **C** Complication

Seven studies (comprising 910 patients) offered data on the operative time of MOSES mode and regular mode holmium laser lithotripsy [12–14, 16–19]. According to the overall synthesis results, the MOSES technique was associated with significantly lower operation time (SMD =  − 0.43; 95% CI − 0.79 to − 0.08, *P* = 0.016, Fig. 2A). However, high heterogeneity was detected among the studies (I^2^ = 82.7%; *P* < 0.001; Fig. 2A). The Additional file 5 (Fig. S1) presented the corresponding funnel plot. After performing the sensitivity analyse no heterogeneity was found in the included studies (Additional file 6: Fig. S2A). We noticed no evidence of Egger's publishing bias (t =  − 1.64; *P* = 0.163). The conclusions of the sub-group analysis are presented in Table 1. The operation time can be greatly shortened by using the Moses mode laser in Asia. The subgroup with a sample size ≤ 100 had a more significant reduction. In the subgroup that included fewer women than men, the MOSES mode laser time was shorter than the regular mode laser and had statistically significant differences and lower heterogeneity (Table 1).

**Table 1:** Subgroup analysis of the association between Moses and Regular modes of holmium laser

Category of variables	Heterogeneity				*P* value for difference
	Studies, n	I^2^, (%)	*P*	Effect (95% CI)	
*Operation time*	5				
Study design					
Randomized	1	–	–	− 0.40 (− 0.87, 0.070)	0.096
Non-randomized	6	85.5	0.000	− 0.45 (− 0.85, − 0.04)	0.031
Geographic area					
Asia	2	68.1	0.077	− 0.74 (− 1.43, − 0.05)	0.035
North America	3	40.9	0.184	− 0.04 (− 0.32, 0.23)	0.752
Europe	2	93.1	0.000	− 0.75 (− 1.90, 0.41)	0.207
Sample size					
≤ 100	3	75.5	0.017	− 0.96 (− 1.61, − 0.30)	0.004
> 100	4	66.9	0.028	− 0.14 (− 0.41, 0.14)	0.339
Mean age					
≤ 50	3	78.2	0.010	− 0.46 (− 0.97, 0.06)	0.081
> 50	4	88.0	0.000	− 0.42 (− 0.99, 0.14)	0.144
Female/male ratio					
< 1	3	88.1	0.000	− 0.87 (− 1.73, − 0.02)	0.045
> 1	3	77.9	0.011	− 0.12 (− 0.50, 0.26)	0.535
Stone size					
< 20 mm	4	82.9	0.000	− 0.35 (− 0.70, 0.01)	0.055
≥ 20 mm	1	–	–	− 1.19 (− 1.95, − 0.44)	0.002
*Efficiency-SFR*	5				
Study design					
Randomized	1	–	–	1.60 (0.41, 6.23)	0.498
Non-randomized	4	37.5	0.156	1.31 (0.72, 2.37)	0.372
Geographic area					
Asia	2	55.7	0.133	2.33 (0.27, 20.05)	0.441
North America	3	0.0%	0.470	0.92 (0.56, 1.51)	0.743
Europe	2	30.5%	0.230	3.12 (0.67, 14.51)	0.147
Sample size					
≤ 100	3	22.3%	0.276	3.58 (0.98, 13.16)	0.055
> 100	4	0.0%	0.637	0.97 (0.64, 1.48)	0.900
Mean age					
≤ 50	4	10.6%	0.327	1.33 (0.68, 2.60)	0.411
> 50	3	47.4%	0.127	1.43 (0.59, 3.47)	0.424
Female/male ratio					
< 1	3	8.1%	0.337	3.68 (1.09, 12.47)	0.036
> 1	3	0.0%	0.559	0.93 (0.60, 1.44)	0.748
Stone size					
< 20 mm	6	13.7%	0.327	1.14 (0.73, 2.78)	0.553
≥ 20 mm	1	–	–	11.88 (0.58, 241.68)	0.107
Stone-free definition					
≤ 2 mm	4	43.8%	0.148	1.21 (0.60, 2.41)	0.596
≤ 4 mm	2	0.0%	0.787	1.38 (0.61, 3.14)	0.445
*Complication*	5				
Study design					
Randomized	1	–	–	0.73 (0.15, 3.51)	0.692
Non-randomized	4	0.0%	0.659	0.86 (0.44, 1.71)	0.675
Geographic area					
Asia	1	–	–	1.16 (0.42, 3.25)	0.773
North America	2	0.0%	0.719	0.91 (0.34, 2.44)	0.852
Europe	2	0.0%	0.794	0.42 (0.11, 1.59)	0.204
Sample size					
≤ 100	2	0.0%	0.563	0.53 (0.17, 1.69)	0.283
> 100	3	0.0%	0.800	1.02 (0.48, 2.15)	0.963
Mean age					
≤ 50	1	–	–	1.16 (0.42, 3.25)	0.773
> 50	4	0.0%	0.655	0.69 (0.31, 1.53)	0.364
Female/male ratio					
< 1	3	0.0%	0.794	0.42 (0.11, 1.59)	0.204
> 1	2	0.0%	0.905	1.12 (0.50, 2.48)	0.783
Stone-free definition					
≤ 2 mm	4	0.0%	0.659	0.86 (0.44, 1.71)	0.675
≤ 4 mm	1	–	–	0.73 (0.15, 3.51)	0.692

### Efficacy of stone-free assessment

In the evaluation of stone-free rates between MOSES and conventional techniques, 910 patients from 7 studies were considered [12–14, 16–19]. Figure 2B showed that there was no statistically significant difference between the two modalities (RR = 1.06; 95% CI 0.99–1.12) (*P* = 0.301) with no significant heterogeneity (I^2^ = 16.8%). The corresponding funnel plot was showed in the Additional file 7 (Fig. S3). The result of sensitivity study was shown in Additional file 6: Fig. S2B. According to the results of Egger’s (t = 0.56; *P* = 0.602), publication bias was not founded. There was also an analysis of subgroups (see Table 1). In the subgroup where there were fewer women than men, the MOSES group had a considerably greater stone-free percentage than the overall group (RR 3.68; 95% CI 1.09–12.47) (*P* = 0.036; Table 1).

### Safety outcome assessment

The complication comparison between MOSES mode and normal mode comprised data from 6 studies (with 779 patients) [12–14, 16–18]. In relation to safety, no significant difference was found in the forest plot (RR = 0.85, 95% CI 0.48–1.53, *P* = 0.806, Fig. 2C) with no heterogeneity (I^2^ = 0.0%). Additional file 8: Fig. S4 showed a funnel graph about complication metrics. No heterogenous study was found in the Additional file 6: Fig. S2C. Egger's findings demonstrated that there was no publication bias (t =  − 2.77, *P* = 0.07). In contrast, the prospective factor subgroup analysis did not reveal any statistically significant associations (see Table 1).

## Discussion

Holmium Laser lithotripsy is currently one of the gold standard treatments for treating symptomatic renal and ureteral stones sized in 1–2 cm^5^. Urologists are pursuing research in two primary ways to improve the efficacy of laser lithotripsy: developing innovative laser sources and/or enhancing the energy delivery of Ho: YAG itself. The most accepted innovation for holmium: yttrium–aluminum-garnet laser lithotripsy is the addition of laser pulse modulation [20].

MOSES technology is a higher-powered Ho: YAG laser pulse modality. It is a mixed pattern comprised of two sub-pulses to first produce a vapor bubble [21]. The vapor bubble provides a pathway for the next sub-pulse to be transferred to the target rather than dissipated in the water [22]. Many reviews have given narrative appraisals of the MOSES mode and recognized its advantages over the regular mode. However, these are lack clinical data and based on in vitro or animal experiments. A modest number of clinical studies have been undertaken in different regions internationally in recent years. This study intends to gather such information to determine the clinical value of the MOSES mode in urinary stone treatment, especially kidney stone and ureteral stones.

In the study conducted by Brenton [23], MOSES technology had the advance of decreased heat production, which is proportional to the power, although it used higher energy. It suggested the safety assurance of MOSES technology in clinical use.

After careful literature searching and data pooling, this meta-analysis found that using MOSES mode could reduce operation time and demonstrated the point above. However, the result was relatively heterogeneous. Moreover, we used subgroup analysis to compare the two modes. In the subgroup with more men than women, the procedure took significantly less time, and there was no heterogeneity in the results. This could be introduced by the different types of stones obtained by men and women differ significantly. Males have a higher probability of having calcium oxalate stones, while females have more chance to acquire infection stones [24, 25]. Magnesium ammonium phosphate (MAP)- and carbonate apatite (CA)-based infection-related stones have a loose structure. As a result, stones in females may be easier to break up, and the procedure may take less time. The MOSES mode laser’s advantages in terms of time cannot be shown. Since there was no detailed stone composition data offered in the included studies, it was impossible to validate this hypothesis.

A previous economy-based study found that procedural time savings did not result in overall cost savings, offset by the MOSES technology's cost [26]. However, short operative times can reduce the incidence of infectious complications based on known predisposing factors [27, 28]. This can increase the average daily surgical volume and lower the risk of infection in the future. The importance of the shortened operation time requires further consideration.

In this meta-analysis, we didn’t find that there was a significant complication difference between MOSES laser and regular laser. There were two main possible reasons. First, we believed that all the cases included in this meta-analysis received proper and sufficient perioperative treatments to avoid any foreseeable complications. Second, we noticed that in all seven included studies, operation times were all in the safe ranges for RIRS [29]. When discussing the possible complication benefits of the MOSES laser within a relatively safe operation time, the required sample size may well exceed the numbers that this meta-analysis can provide since the reported complication rate after the RIRS was low. This result also indicated that the MOSES laser may be able to bring significant operative time and complication benefits in the RIRS treatment of large-volume kidney stones or renal cast stones.

MOSES technique can achieve in-situ gravel. The stones have a smaller possibility to drift or escape. High power and high-frequency holmium laser can be more efficient in powdering kidney stones so that no stone remains. Kristian M. B. found that increasing the pulse energy parameters for the MOSES distance mode might lower the size of pieces. The meta-analysis revealed no statistically significant difference between the stone-free rates of MOSES mode and standard mode. According to the results of the trim-and-fill analysis, three studies appear to be missing. After filling in the missing data, the findings revealed no significant difference between the two procedures. This analysis validated the stability of our result. Although current evidence suggests that the MOSES laser may not deliver significant benefits in SFR, the operative time benefit from MOSES appears to be able to compensate for this regrettable point. Operation time is always one of the major concerns for urologists during RIRS surgery. Excessive surgical duration is accompanied by an increased risk of postoperative complications. Therefore, when the ideal powdering or stone basketing couldn’t be achieved within an ideal time, urologists often choose a secondary surgery to ensure patient safety. According to the current findings, using MOSES results in a similar stone clearance rate in a shorter operative time, showing a higher efficiency. When assuming the stone burden is the same and applying the MOSES laser, an energy system that can bring significant gains in stone fragmentation efficiency, urologists will have more time to perform stone extracting work. If the surgeon is more patient and spends more time with the patient, the stone-free rate should be higher.

There were still some limitations in this study. The meta-analysis included only one RCT and six cohort studies, which might introduce bias. Furthermore, the parameters of holmium laser were different in included studies, which might cause heterogeneity. Some studies didn’t refer to the composition of the stones in patients, which could lead to significant heterogeneity. Studies, especially RCTs with greater sample sizes, are required to derive more reliable results.

## Conclusion

According to the meta-analysis based on all available published data, the MOSES mode laser was superior to the regular mode laser in procedure time. Furthermore, there was no noticeable difference between the two treatment modes in terms of stone removal rates or the incidence of complications. Additional clinical investigations may indicate that the MOSES system can increase SFR. When finances allow, the MOSES laser modality might be a good option. The findings of this study should, however, be interpreted with care due to the limitations noted above.

## Supplementary Information

**Additional file 1: Table S1.** The search strategies used in the databases.

**Additional file 2:** **Table S2**. Basic information of included studies in this meta-analysis.

**Additional file 3: Table S3**. Newcastle-Ottawa scale score of the reviewed studies.

**Additional file 4: Table S4**. Jadad score for RCT.

**Additional file 5: Figure S1**. Funnel plot of operative time.

**Additional file 6: Figure S2.** Sensitivity analyses of included studies. A Operative time. B Stone-free rate. C Complication.

**Additional file 7: Figure S3**. Funnel plot of stone-free rate.

**Additional file 8: Figure S4**. Funnel plot of complication.

## Data Availability

All data generated or analyzed during this study are included in this published article and its Additional files.
