# Supplementary material for: Comparison of the efficacy and safety of holmium laser with the Moses technology and regular mode for stone treatment: a systematic review and meta-analysis
Source: BMC Urol. 2023 May 30;23:99. doi: 10.1186/s12894-023-01264-z (PMC10230678; doi:10.1186/s12894-023-01264-z)
Supplement: Supplementary file 1 — Additional file 1: Table S1. The search strategies used in the databases. [file 12894_2023_1264_MOESM1_ESM.docx]

**Table S1: The search strategies used in the databases.**

| PubMed | |
| --- | --- |
| #1 | Search: **lithotripsy[Title/Abstract]** Sort by: **Publication Date** |
| #2 | Search: **laser[Title/Abstract]** Sort by: **Publication Date** |
| #3 | Search: **holmium laser[Title/Abstract]** Sort by: **Publication Date** |
| #4 | Search: **Holmium YAG Lasers[Title/Abstract]** Sort by: **Publication Date** |
| #5 | Search: **Lasers, Ho YAG[Title/Abstract]** Sort by: **Publication Date** |
| #6 （#2 OR #3 OR #4 OR #5） | Search: **(((laser[Title/Abstract]) OR (holmium laser[Title/Abstract])) OR (Holmium YAG Lasers[Title/Abstract])) OR (Lasers, Ho YAG[Title/Abstract]** Sort by: **Publication Date** |
| #7 | Search: **Moses[Title/Abstract]** Sort by: **Publication Date** |
| #8 （#1 AND #6 AND #7） | Search: **((lithotripsy[Title/Abstract]) AND ((((laser[Title/Abstract]) OR (holmium laser[Title/Abstract])) OR (Holmium YAG Lasers[Title/Abstract])) OR (Lasers, Ho YAG[Title/Abstract]))) AND (Moses[Title/Abstract])** Sort by: **Publication Date** |
| #9 | Search: **(randomized controlled trial[Publication Type]) OR (prospective[Title/Abstract]) OR (retrospective[Title/Abstract]) OR (cohort study[Title/Abstract])** Sort by: **Publication Date** |
| #10 (#8 AND #9) | Search: **(((lithotripsy[Title/Abstract]) AND ((((laser[Title/Abstract]) OR (holmium laser[Title/Abstract])) OR (Holmium YAG Lasers[Title/Abstract])) OR (Lasers, Ho YAG[Title/Abstract]))) AND (Moses[Title/Abstract])) AND ((randomized controlled trial[Publication Type]) OR (prospective[Title/Abstract]) OR (retrospective[Title/Abstract]) OR (cohort study[Title/Abstract]))** Sort by: **Publication Date** |

| **Embase** | |
| --- | --- |
| #1 | **'moses':ab,ti** |
| #2 | **'lithotripsy':ab,ti** |
| #3 | **'holmium laser':ab,ti OR 'laser':ab,ti OR 'holmium yag lasers':ab,ti OR 'ho yag':ab,ti** |
| #4 | **'randomized controlled trial'/exp OR 'prospective study'/exp OR 'retrospective study'/exp OR 'cohort study'/exp** |
| #5 | **#1 AND #2 AND #3 AND #4** |

| **Web of science** | |
| --- | --- |
| #1 | **TS=(moses)** |
| #2 | **((TS=(lithotripsy)) OR TS=(Retrograde intrarenal surgery)** |
| #3 | **(((TS=(laser)) OR TS=(holmium laser)) OR TS=(holmium yag lasers)) OR TS=(Lasers, Ho YAG)** |
| #4 | **(((TS=(randomized controlled trial)) OR TS=(prospective study)) OR TS=(retrospective study)) OR TS=(cohort study)** |
| #5 | **#1 AND #2 AND #3 AND #4** |

| **Cochrane library** | |
| --- | --- |
| #1 | MeSH descriptor: [Lithotripsy] explode all trees |
| #2 | MeSH descriptor: [Holmium] explode all trees |
| #3 | moses |
| #4 | #1 AND #2 AND #3 |

| **CNKI** | |
| --- | --- |
| #1 | Moses |
| #2 | Calculi OR Stone |
| #3 | Randomized controlled trial OR Retrospective study OR Prospective study |
| #4 | #1 AND #2 AND #3 |
