# Supplementary material for: Comparison of the efficacy and safety of holmium laser with the Moses technology and regular mode for stone treatment: a systematic review and meta-analysis
Source: BMC Urol. 2023 May 30;23:99. doi: 10.1186/s12894-023-01264-z (PMC10230678; doi:10.1186/s12894-023-01264-z)
Supplement: Supplementary file 2 — Additional file 2: Table S2. Basic information of included studies in this meta-analysis. [file 12894_2023_1264_MOESM2_ESM.docx]

Table S2

Table S2. Basic information of included studies in this meta-analysis.

| Study | Year | Country | Study design | Sample size (Control/Intervention) | Mean age (Control/Intervention) | Female/male | Mean stone size (mm) (Control/ Intervention) | Intervention | Control | Control-type | Using ureteral sheath (Control/Intervention) | Stone extracting technique | Operation time outcome assessment | Efficacy outcome assessment | Safety outcome assessment | Quality assessment* |
| --- | --- | --- | --- | --- | --- | --- | --- | --- | --- | --- | --- | --- | --- | --- | --- | --- |
| Ibrahim A | 2020 | Canada | RCT | 36/36 | 54.7±13.6/ 57.4±11.9 | NR | 14±9.7/ 17±15 | Moses mode | Regular mode | Lumenis PulseTM 120H;  An energy level of 0.4 J and rate of 80Hz for stone pulverization (Stone Dusting) and 1.0 J and rate of 10 Hz for stone fragmentation | 14 (38.9%)/ 11 (30.6%) | Ureteral stones  were fragmented and basketed out while renal stones were pulverized | Procedural time was measured as the time from introduction of the ureteroscope till the final removal of the ureteroscope | Success rate was assessed using low dose CT scans at 3 months. Success rate was defined as no ureteral fragments or presence of non‐obstructive renal fragments of ≤4 mm | Recorded intraoperative complications | 7 |
| Knoedler M. A. | 2022 | USA | RCS | 66/110 | 56.8±14.8/ 58.7±13.2 | 90/86 | 11.6±9.2/ 11.8±7.9 | Moses mode | Regular mode | Lumenis PulseTM P120H holmium laser system;  Moses D/F/L 200-micron fiber on the Moses mode or the standard 200-micron fiber on the regular mode 0.3 J and 80 Hz for dusting and 0.8 J and 8 Hz for fragmentation | 4 (4.5%)/ 7 (6.3%) | Laser stones to submillimeter fragments for proximal ureteral and renal stones, while distal ureteral stones are treated with a combination of laser fragmentation and basket extraction | Procedural time was measured from the time the cystoscope was inserted into the patient to the time all instruments were removed from the patient | Stone free was defined as no stone fragments >2 mm on postoperative imaging | Recorded postoperative complications;  Complications included any patient requiring unplanned admission for any reason within 30 days of surgery | 6 |
| Pietropaolo A. | 2021 | UK | RCS | 38/38 | 58.1±14.5/ 53.8±5.8 | 30/46 | 16.5 ± 11.3/ 15.5 ± 9.9 | Moses 60W laser | Holmium 20 W laser | a Moses 60W laser: 0.4–0.8 J, 20–35 Hz with Moses setting; Holmium 20 W laser: 0.4–0.8 J, 12–18 Hz | 21 (55.2%)/22 (57.8%) | NR | Operative time | Stone free rate (SFR) was defined as complete clearance of stones endoscopically and ≤2 mm fragments on post-operative imaging done 2–4 months later | Recorded postoperative complications | 6 |
| Wang M | 2021 | China | RCS | 102/114 | 47.97±11.55/ 49.91±12.86 | 109/107 | 12.00/12.00 | Moses mode | Regular dusting mode | Using a holmium laser lithotripter (Lumenis® PulseTM P120H, Israel) and MOSES SlimLine 200-μm fibers or Lumenis SlimLine D/F/L 200-μm fibers, 2 different laser modes (MCM or RDM) were used, respectively, for lithotripsy. | All | Stone retrieval baskets were not used in all operations | Operative time | SFR was defined as 2U or 2X, having complete absence of stones or clinically insignificant fragments ≤2 mm on abdominal ultrasound or XR for kidney, ureter, and bladder | Recorded postoperative complications which included fever, acute renal failure (ARF) | 6 |
| Mai H | 2022 | China | RCS | 16/16 | 45.2±15.8/ 42.9±15.0 | 9/23 | 24±2.0/ 25±3.0 | Moses holmium laser + FURL group | Holmium laser + FURL group | Lumenis PulseTM P120 H;  An energy level of 0.3-0.4 J and rate of 80 Hz, laser output power of 24-32W for moses group; An energy level of 0.6-0.8 J and rate of 40 Hz, laser output power of 24-32W for regular group | All | Stone retrieval baskets were used after lithotripsy | Operative time | The stone removal criteria were the absence of visible stones in the CT scan | Recorded postoperative complications | 6 |
| Pietropaolo A. | 2022 | UK | PCS | 168/39 | 53.94±16.03/ 44.64±24.68 | 79/128 | 9.69±2.47/ 13.09±7.95 | Moses 60W laser | Holmium 20 W laser | Using a 60W Holmium moses integrated laser, 0.4–1J and 20–50Hz for moses group; 20W holmium laser, a maximum of 0.4–1J and 10–  18Hz for regular group | 91 (54.1%)/ 15 (38.4%) | Fragments were  retrieved using Cook Ngage stone extractor | Operative time | Stone free rate was defined as complete clearance of stones  endoscopically and ⩽2mm fragments on postoperative imaging | All  intra- and postoperative complications were recorded | 6 |
| Harris W. N. | 2022 | USA | RCS | 86/45 | 12.75±4.00/ 13.30±3.58 | 69/62 | 10.65±5.53/ 11.10±8.10 | Moses mode | Holmium 30 W laser | Lumenis  120 W MOSES holmium laser, 0.8e1 J and 10e12 Hz with MOSES contact  or distance in the kidney and 0.6 J and 8 Hz in the ureter for  fragmentation; Dornier 30 W holmium:YAG laser for regular group | NR | NR | Operative time was defined by  procedure start and end times listed in each perioperative  record | Stone  clearance was assessed by post-operative ultrasound with  clearance defined as calculi <4 mm | NR | 4 |

RCS, retrospective cohort study; RCT, randomized control trail; PCS, prospective cohort study; FURL, flexible ureteroscopic lithotripsy; NR, not reported. *, Jadad scores for RCT and NOS score for non-randomized studies
