# Supplementary material for: Comparison of the efficacy and safety of holmium laser with the Moses technology and regular mode for stone treatment: a systematic review and meta-analysis
Source: BMC Urol. 2023 May 30;23:99. doi: 10.1186/s12894-023-01264-z (PMC10230678; doi:10.1186/s12894-023-01264-z)
Supplement: Supplementary file 3 — Additional file 3: Table S3. Newcastle-Ottawa scale score of the reviewed studies. [file 12894_2023_1264_MOESM3_ESM.docx]

Table S3. Newcastle-Ottawa scale score of the reviewed studies

| Study | Selection (4 stars) | | | | Comparability (2 stars) | Outcome (3 stars) | | | Total score |
| --- | --- | --- | --- | --- | --- | --- | --- | --- | --- |
|  | Representativeness score | Selection of the non exposed cohort | Ascertainment of exposure | Demonstration that outcome of interest was not present at start of study | Comparability of cohort on the basis of the design or analysis | Assessment of outcome | Was follow-up long enough for outcomes to occur? | Adequacy of follow up of cohort |  |
| Knoedler M. A.  (2022) | ★ | ★ | ★ | ★ | ★ | ★ | ★ | / | 7 |
| Pietropaolo A.  (2021) | ★ | ★ | ★ | ★ | ★ | ★ | / | ★ | 7 |
| Wang M (2021) | ★ | ★ | ★ | ★ | ★ | ★ | / | ★ | 7 |
| Mai H (2022) | ★ | ★ | ★ | ★ | ★ | ★ | / | ★ | 7 |
| Pietropaolo A. (2022) | ★ | ★ | ★ | ★ | ★ | ★ | / | / | 6 |
| Harris W. N. (2022) | / | ★ | ★ | ★ | ★ | ★ | ★ | / | 6 |
