# Supplementary material for: Comparison of the efficacy and safety of holmium laser with the Moses technology and regular mode for stone treatment: a systematic review and meta-analysis
Source: BMC Urol. 2023 May 30;23:99. doi: 10.1186/s12894-023-01264-z (PMC10230678; doi:10.1186/s12894-023-01264-z)
Supplement: Supplementary file 4 — Additional file 4: Table S4. Jadad score for RCT. [file 12894_2023_1264_MOESM4_ESM.docx]

**Table S4: Jadad score for RCT.**

| **Study** | **Design** | **Randomization** | **Double blinding** | **Follow-up** | **Total points/rank** |
| --- | --- | --- | --- | --- | --- |
| Ibrahim A (2020) | RCT | 4 | 2 | 1 | 7/High |
