# Supplementary figures and images for: Comparison of the efficacy and safety of holmium laser with the Moses technology and regular mode for stone treatment: a systematic review and meta-analysis
Source: BMC Urol. 2023 May 30;23:99. doi: 10.1186/s12894-023-01264-z (PMC10230678; doi:10.1186/s12894-023-01264-z)

Funnel plot with pseudo 95% confidence limits

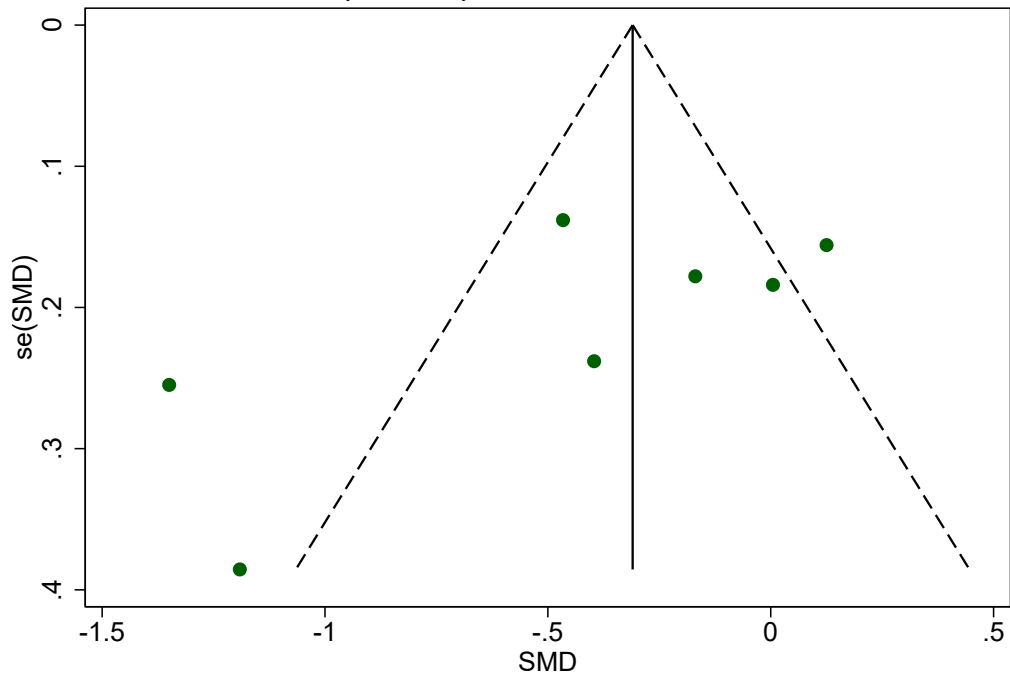

Supplement: Supplementary file 5 — Additional file 5: Figure S1. Funnel plot of operative time. [file 12894_2023_1264_MOESM5_ESM.pdf]

A

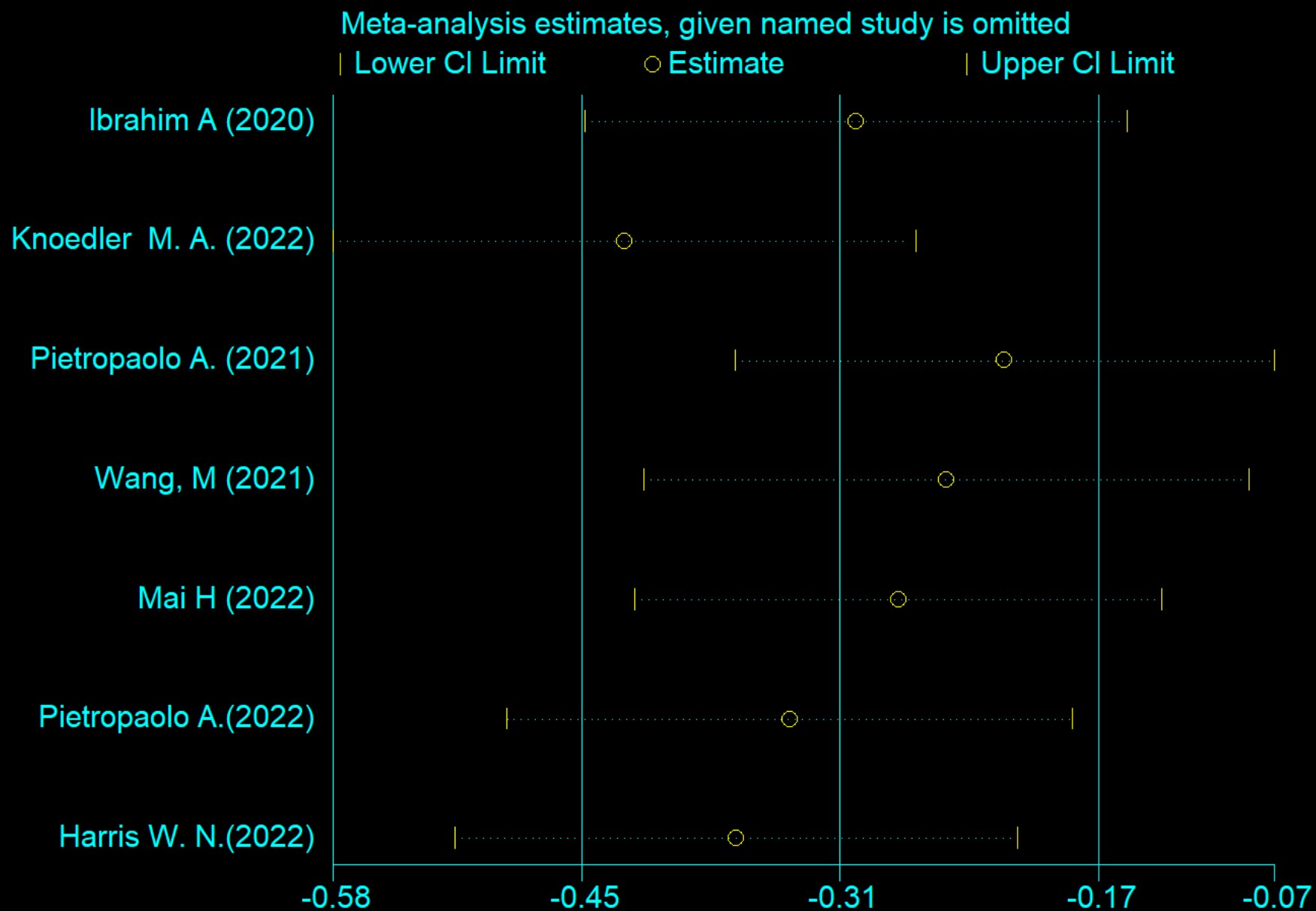

B

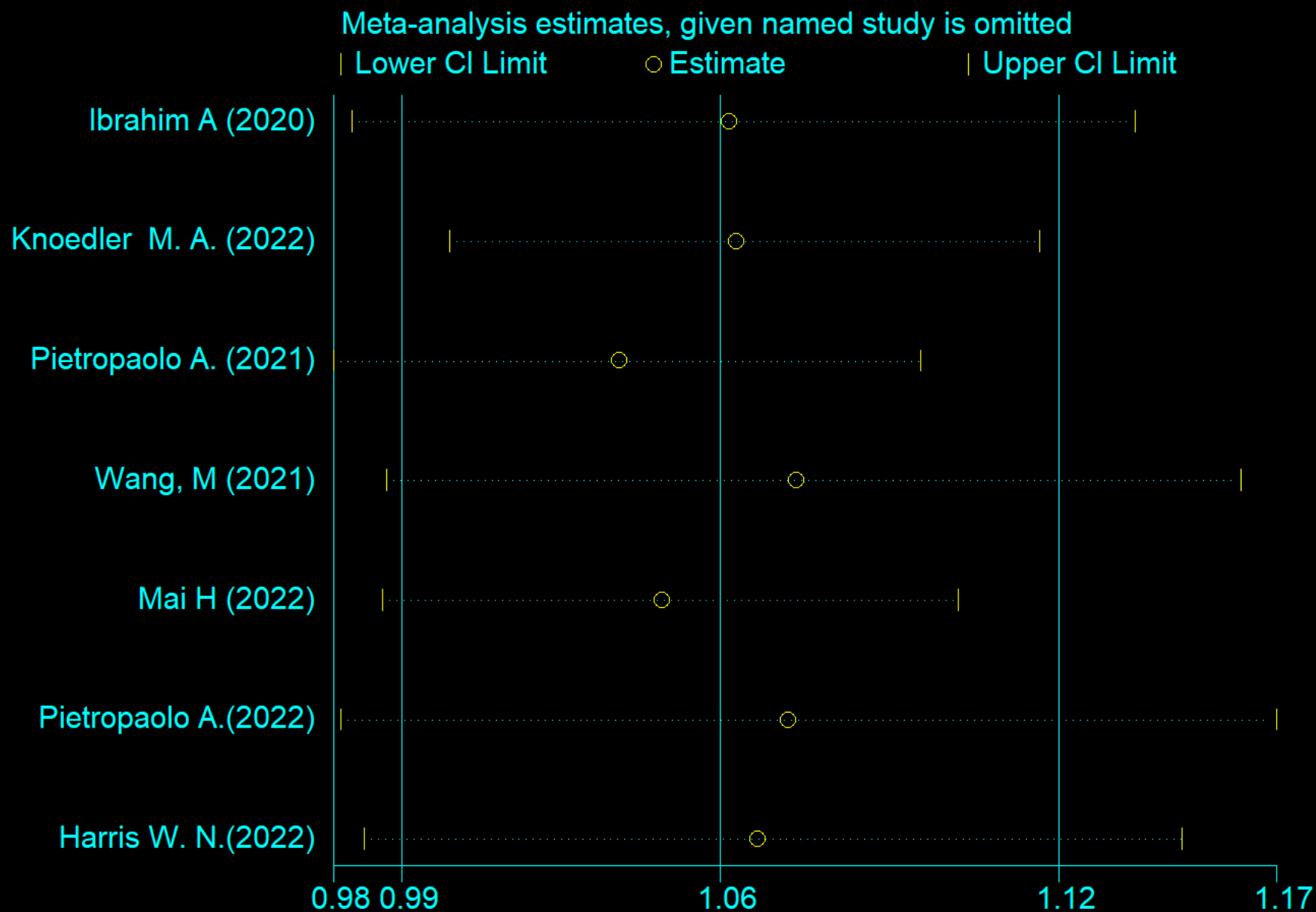

C

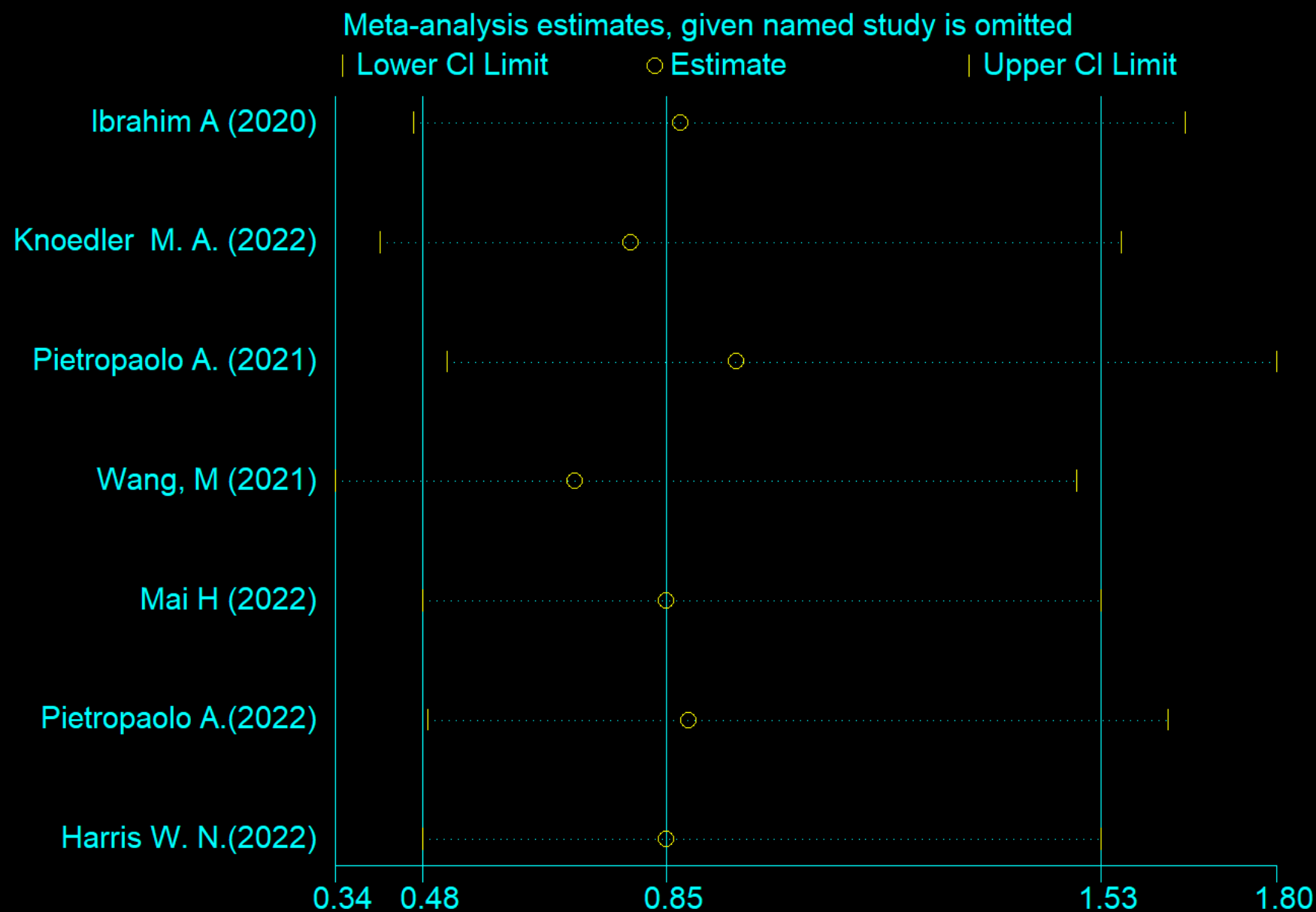

Supplement: Supplementary file 6 — Additional file 6: Figure S2. Sensitivity analyses of included studies. A Operative time. B Stone-free rate. C Complication. [file 12894_2023_1264_MOESM6_ESM.pdf]

Funnel plot with pseudo 95% confidence limits

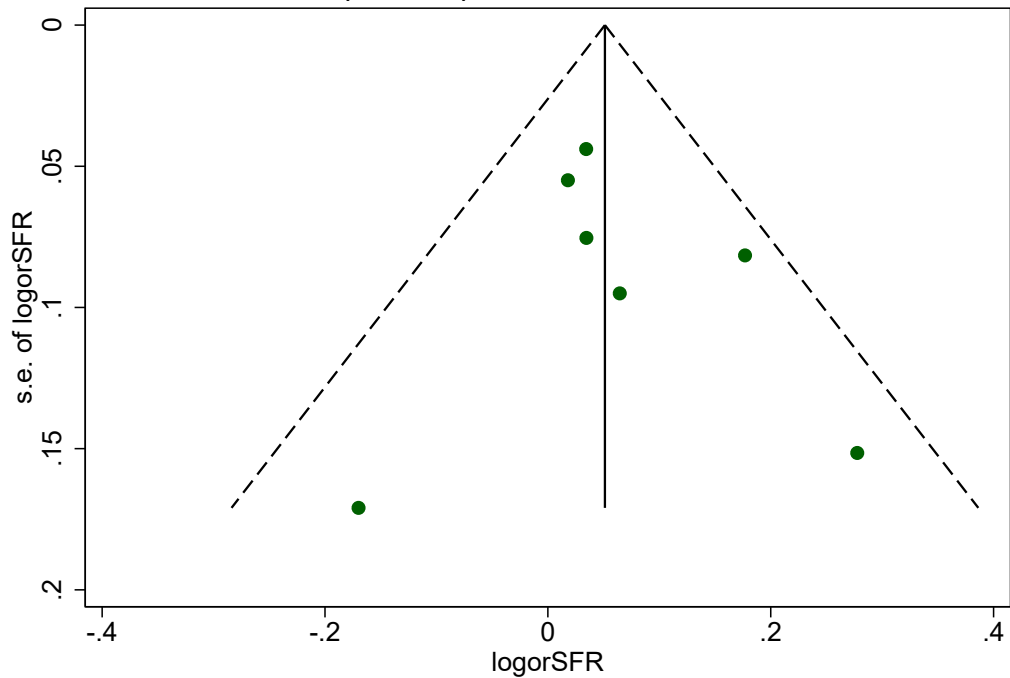

Supplement: Supplementary file 7 — Additional file 7: Figure S3. Funnel plot of stone-free rate. [file 12894_2023_1264_MOESM7_ESM.pdf]

Funnel plot with pseudo 95% confidence limits

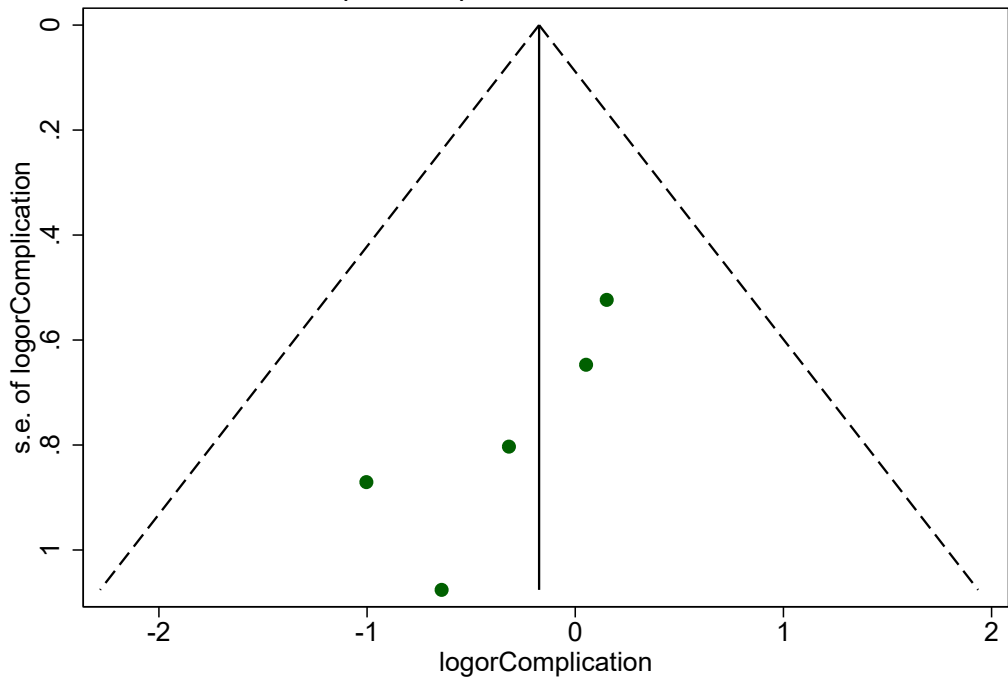

Supplement: Supplementary file 8 — Additional file 8: Figure S4. Funnel plot of complication. [file 12894_2023_1264_MOESM8_ESM.pdf]
